# Natural Killer Cells and Their Activation Status in Normal Pregnancy

**DOI:** 10.1155/2013/906813

**Published:** 2013-03-27

**Authors:** Beatrice Mosimann, Marion Wagner, Hassan Shehata, Leona C. Y. Poon, Brian Ford, Kypros H. Nicolaides, Amolak S. Bansal

**Affiliations:** ^1^Harris Birthright Research Centre for Fetal Medicine, King's College Hospital, London SE5 9RS, UK; ^2^Department of Obstetrics and Gynaecology, St. Helier Hospital, Carshalton SM5 1AA, UK; ^3^Department of Obstetrics and Gynaecology, St. Mary's Hospital, Imperial College Healthcare NHS Trust, London, UK; ^4^Department of Immunology, St Helier Hospital, Carshalton, Surrey SM5 1AA, UK; ^5^Department of Fetal Medicine, University College Hospital, London WC1E 6DB, UK

## Abstract

Increased peripheral blood-activated NK cell counts are associated with increased risk of miscarriage and failed in vitro fertilization treatment. However, assessment of activated peripheral NK cells in normal and pathological pregnancies beyond implantation and early miscarriage has not been described. Total CD69 expressing NK cells counts were measured by flow cytometry in healthy women with singleton pregnancies, including 45 at 11^+6^–13^+6^ weeks' gestation, 46 at 20^+0^–22^+4^ weeks, and 42 at 31^+6^–33^+5^ weeks. The number of peripheral blood NK cells decreased, whereas the percentage of activated CD69 expressing NK cells increased from the first to the third trimester of pregnancy. This study shows the course of peripheral blood NK cells and activated CD69 expressing NK cells in uncomplicated nulliparous singleton pregnancies. This is a first step in understanding their implication in pathological pregnancies.

## 1. Introduction

It is now accepted that for a normal pregnancy to occur, the maternal immune system must be rendered more tolerant towards the semiallogeneic fetus. The required changes must ultimately result in controlled modulation of the uterine natural killer (uNK) cells that represent the most abundant cell population at the fetomaternal interface. These cells play a critical role in implantation and particularly in vascular remodelling and trophoblast invasion [1, 2]. As such NK cells have a critical role in the healthy progression of the pregnancy by maintaining the balance between placental function and fetal requirements. 

NK cells are also found in the peripheral blood and like uNK cells, these may be recognized by the expression of cell surface markers. They are usually CD3 negative, but express CD16 and CD56. NK cells are subdivided, by the intensity of expression of CD56, into a CD16+CD56^bright^ and CD16+CD56^dim^. Peripheral blood NK (pNK) cells are predominantly CD16+CD56^dim^, whereas uNK cells are predominantly CD16+CD56^bright^ [3, 4]. The exact relationship between these two sub-groups of NK cells is unclear but it has been suggested that uNK migrates from the systemic vascular system [5, 6]. Importantly, however, the factors that regulate uterine and peripheral blood NK cells are likely similar. Thus assessing the level of activation of the peripheral blood NK cells gives information of the state of the uterine cells [7].

CD69 is one of the earliest specific markers of NK cell activation [8–10]. Activated NK cells release cytokines that activate other NK cells and the cellular immune system generally [10]. Elevated NK cell CD69 expression is associated with increased cytotoxicity and target cell lysis [11, 12]. In normal pregnancy, compared to an anembryonic pregnancy, NK cell cytotoxicity is decreased suggesting that activated CD69 expressing NK cells play an important role in the control of trophoblast growth and placental development [13]. Supportive evidence is provided by in vitro models which demonstrated that activated CD69 positive NK cells are capable of lysing trophoblasts [14, 15]. There is evidence that increased pNK cells and activated NK cells are associated with increased risk of miscarriage and failed in vitro fertilization (IVF) treatment [16–19]. Moreover, immunomodulatory therapies that aim to reduce the numbers of these activated NK cells have shown benefit with improved outcomes [20]. However, the value of assessing activated pNK cells in the investigation of pregnancy complications beyond implantation and early miscarriage has not been described.

The aims of this pilot study were firstly, to examine if the number of activated NK cells changes with the progression of a normal pregnancy and in particular to observe variations between each trimester of pregnancy and secondly, to define a provisional normal range of peripheral blood total CD16/56 NK cells and their CD69 expressing activated subset as a first step for the investigation of these parameters in pathological pregnancies. 

## 2. Material and Methods

This was a prospective cross-sectional observational study of consecutive nulliparous pregnant women attending for their routine antenatal visits in the first, second, and third trimesters of pregnancy between October 2011 and February 2012 at King's College Hospital, London. Written informed consent was obtained from the women agreeing to participate in the study, which was approved by King's College Hospital Ethics Committee. 

Blood (6 mL) obtained from the antecubital vein was collected into heparinized tubes from a total of 133 healthy women with singleton pregnancies, including 45 at 11^+6^–13^+6^ weeks' gestation, 46 at 20^+0^–22^+4^ weeks, and 42 at 31^+6^–33^+5^ weeks. Analysis of the samples was carried out within 6 hours of collection.

### 2.1. Sample Analysis

This is the first study to assess the variation in numbers of activated NK cells across gestation in normal pregnancy. We have therefore estimated the sample size from our previous work on activated NK cells in recurrent failed IVF [18]. 

Total lymphocyte and total CD16/56 NK cell counts were measured in all the 133 women. Due to purchasing and shipping difficulties of the reagents, CD69 activation marker on the NK cells was only measured in the last 90 cases and there was no selection bias. CD69 was chosen to indicate NK cell activation as our previous unpublished work had shown it to be one of the earliest and probably most robust activation marker of NK cell activation. Additionally we found it to be altered little by several hours of sample storage. This was important as the patients in our study were seen at another hospital and the transit time was up to 5 hours. 

**Figure 1:**
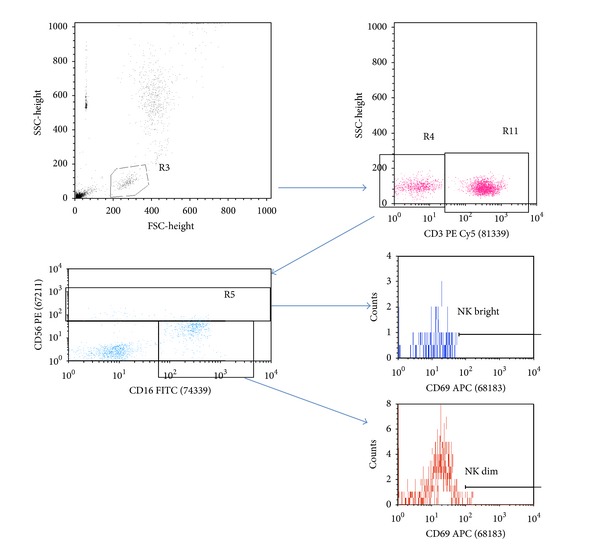
Gating strategy for NK CD69 enumeration: lymphocytes were identified using FSC/SSC and the CD3 negative population was identified. They were further classified into CD16 positive and either CD56 dim or CD56 bright. Each of these populations was then interrogated for CD69 expression.

A lyse no-wash protocol was used to stain the whole blood sample. 10 *μ*L of anti-CD56PE (BD Pharmingen), 10 *μ*L of anti-CD16 FITC (BD Pharmingen), 10 *μ*L of anti-CD3 PE Cy5 (BD Pharmingen), and 10 *μ*L of anti-CD69 APC (BD Pharmingen) were added into a Falcon (BD Biosciences) tube. An isotype control tube containing anti-CD3, CD16, and CD56 but replacing the anti-CD69 with an isotype control antibody (IgG1k, BD Pharmingen) was included for every patient. 50 *μ*L of well-mixed whole blood was then added and tubes were incubated for 15 minutes in the dark. 1 mL of Easylyse (BD Biosciences) was added and tubes were incubated in the dark for a further 15 minutes. Finally 50 *μ*L of Cytocount beads (Dako) was added and the tubes were gently vortexed. Tubes were analyzed on a Fascalibur (BD Biosciences) using Cellquest Pro software. 10,000 beads were counted which correlates with 20,000 to 30,000 lymphocytes depending on the absolute lymphocyte count. The isotype control was used to set the cutoff for the negative population (Figure 1).

### 2.2. Statistical Analysis

The characteristics of the study population are presented in median and range for continuous variables and in number (%) for categorical variables.

Normality of distribution was assessed using probability plots and the Kolmogorov-Smirnov test. Total lymphocyte, total CD16/56 NK cell and CD69 counts were normally distributed (*P* = 0.900; *P* = 0.120; *P* = 0.799). The NK cell count was expressed as a percentage of the total lymphocyte count (NK cell/lymphocyte). CD69 was expressed as a percentage of NK cell count (CD69/NK cell). The mean and 95% confidence interval of lymphocyte, NK cell, NK cell/lymphocyte, CD69, and CD69/NK cell in each trimester were presented in error bar plots. ANOVA with posthoc LSD test was used to compare the mean values between the first and the second trimester, between the second and third trimester, and between the first and the third trimester of pregnancy. 

The statistical software package SPSS 20.0 (SPSS Inc., Chicago, IL) was used for data analysis.

## 3. Results and Discussion

### 3.1. Results

The maternal characteristics of the study population are presented in Table 1. 

**Figure 2:**
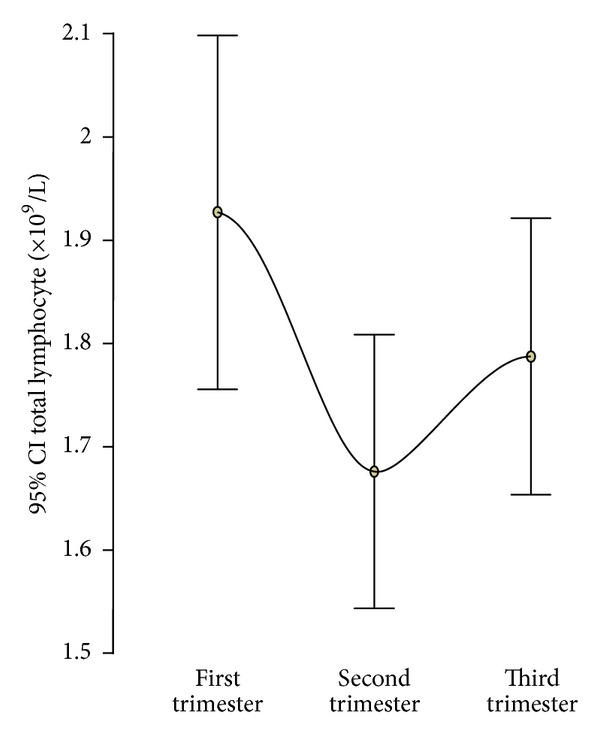
Error bar plot of the mean and 95% confidence interval of total lymphocyte count in each trimester of pregnancy.

The mean (SD) of total lymphocyte count was 1.93 × 10^9^/L (0.57 × 10^9^/L) in the first trimester, 1.68 × 10^9^/L (0.41 × 10^9^/L) in the second trimester, and 1.79 × 10^9^/L (0.43 × 10^9^/L) in the third trimester (Table 2). There was a significant decrease in the total lymphocyte count from the first to the second trimester (*P* = 0.015) and no significant change from the second to the third trimester (*P* = 0.286; Figure 2). There was no significant difference in the mean total lymphocyte count between Caucasian and Afro-Caribbean women in each trimester of pregnancy (*P* = 0.484, *P* = 0.295, *P* = 0.586). In both racial groups, the changes in the total lymphocyte count across each trimester were similar to the total study population. Maternal age had no impact on the total lymphocyte count.

**Figure 3:**
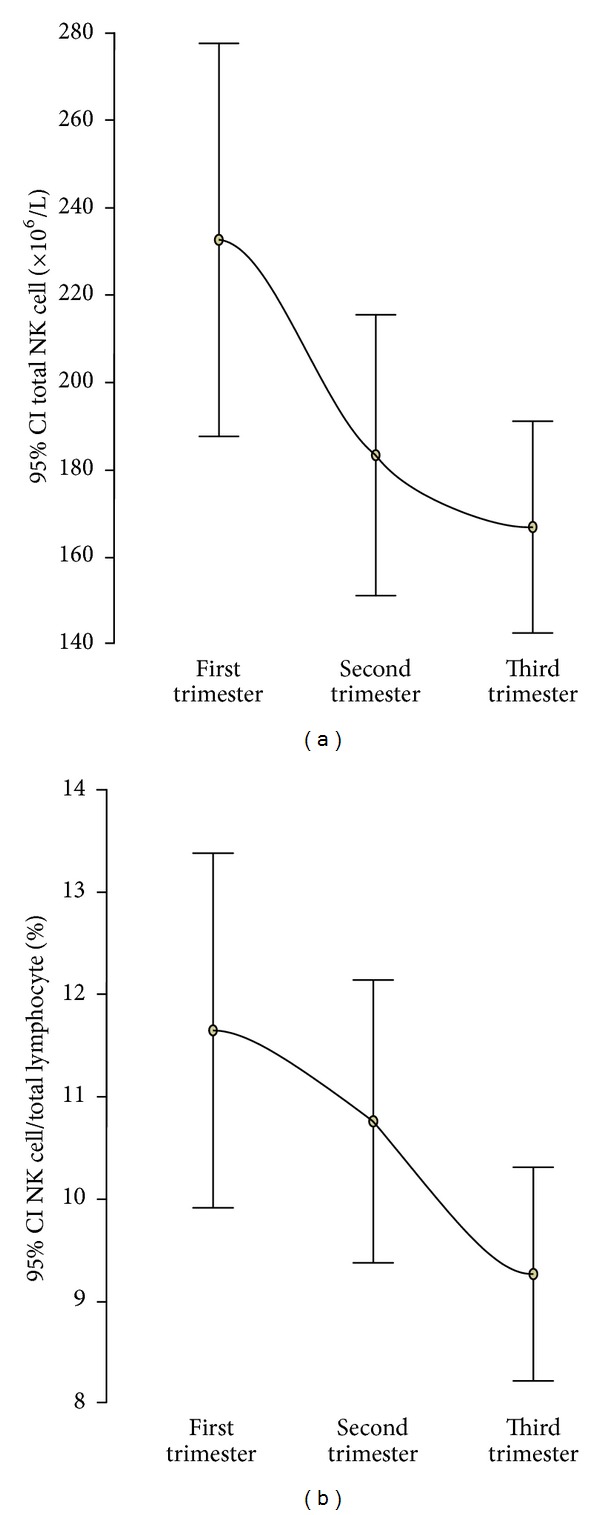
Error bar plots of the mean and 95% confidence interval of total natural killer (NK) cell count (a) and NK cell/total lymphocyte count (%) (b) in each trimester of pregnancy.

The distributions of the total CD16/56 NK cells are illustrated in Figure 3. The mean (SD) of total CD16/56 NK cells was 232.6 × 10^6^/L (149.5 × 10^6^/L) in the first trimester, 183.4 × 10^6^/L (108.1 × 10^6^/L) in the second trimester, and 166.9 × 10^6^/L (77.7 × 10^6^/L) in the third trimester (Table 2). There was a significant decrease in the total NK cell count from the first to the second trimester (*P* = 0.045) and no significant change from the second to the third trimester (*P* = 0.508). Overall there was a significant decrease in both the total NK cell count (*P* = 0.009) and NK cell/lymphocyte (*P* = 0.020) from the first to the third trimester of pregnancy (Figure 3). There was no significant difference in the mean total NK cell count between Caucasian and Afro-Caribbean women in each trimester of pregnancy (*P* = 0.895, *P* = 0.300, *P* = 0.661). 

**Figure 4:**
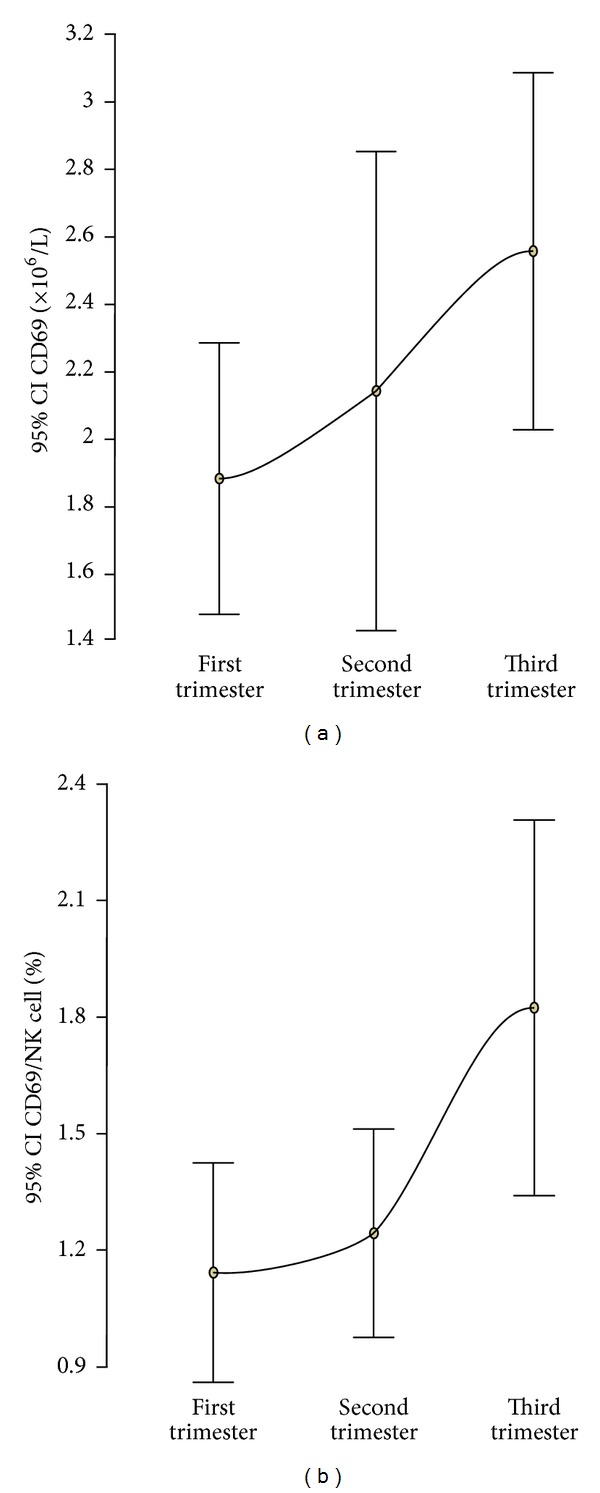
Error bar plots of the mean and 95% confidence interval of CD69 count (a) and CD69/natural killer cell count (%) (b) in each trimester of pregnancy.

The mean (SD) of CD69 activation marker on NK cells was 1.88 × 10^6^/L (1.08 × 10^6^/L) in the first trimester, 2.14 × 10^6^/L (1.90 × 10^6^/L) in the second trimester, and 2.56 × 10^6^/L (1.42 × 10^6^/L) in the third trimester (Table 2). The mean CD69 showed no significant change from the first to the third trimester (*P* = 0.086; Figure 3). However, the CD69/NK cell (%) showed a significant increase from the first to the third trimester (*P* = 0.007; Figure 4). There was no significant difference in the mean CD69 between Caucasian and Afro-Caribbean women in each trimester of pregnancy (*P* = 0.498, *P* = 0.114, *P* = 0.051). 

Both subsets of CD16+CD56^bright^ and CD16+CD56^dim^ express the activation marker CD69. There was a significant reduction in the CD16+CD56^dim^ from the first to the third trimester (205.2 × 10^6^/L, [SD 119 × 10^6^/L] versus 149.2 × 10^6^/L, [SD 84.64 × 10^6^/L]; *P* = 0.039) but not in the CD16+CD56^bright^ (14.4 × 10^6^/L [SD 7.1 × 10^6^/L] versus 14.9 × 10^6^/L [SD 8.9 × 10^6^/L]; *P* = 0.786), and therefore, the overall reduction in NK cells from the first to the third trimester is from the CD16+CD56^dim^. There was a significant increase in the CD16+CD56^bright^ NK cells of CD69 activation marker from the first to the third trimester (0.37 × 10^6^/L [SD 0.29 × 10^6^/L] versus 0.69 × 10^6^/L [SD 0.53 × 10^6^/L]; *P* = 0.002), but not in the CD16+CD56^dim^ NK cells (1.55 × 10^6^/L [SD 1.04 × 10^6^/L] versus 1.85 × 10^6^/L [SD 1.11 × 10^6^/L]; *P* = 0.424), and therefore, the overall increase in CD69/NK cell across gestation is from CD69 on CD16+CD56^bright^ NK cells.

### 3.2. Discussion

This study of normal singleton nulliparous pregnancies without a history of miscarriages has demonstrated that the total lymphocyte count decreased between the first and second trimesters of pregnancy with a non-significant increase from the second to the third trimester. The number of peripheral blood NK cells decreases whereas the percentage of activated CD69 expressing NK cells increases from the first to the third trimester of pregnancy. Monitoring these variables in the same women over their individual pregnancies proved logistically difficult as the samples needed to be transported to the laboratory and analysed within 6 hours. While this would have provided more robust data, we nevertheless feel that our results are sufficiently sound to indicate genuine changes. Comparing these results with women suffering preterm labour, preeclampsia, and other pregnancy-related problems would be an interesting area of research. 

Our findings on the changes in total lymphocyte count with gestation are comparable with those of previous studies. Valdimarsson et al. [21] examined 77 women longitudinally throughout pregnancy and reported that the total lymphocyte count decreased with gestational age to reach a nadir at 25–28 weeks and increasing thereafter until term. Similarly, Lurie et al. [22] examined 726 women longitudinally between the 5th and 41st week of gestation and reported that the total lymphocyte count decreased between the first and second trimesters and then increased in the third trimester. Our finding of a decrease in the number of peripheral blood NK cells with gestational age is contradictory to that of a previous longitudinal study in 23 pregnant women which reported no significant change with gestation [23]. There are also two studies which compared peripheral blood NK cell counts in women during the third trimester of pregnancy to nonpregnant controls and reported that the counts were significantly reduced during pregnancy [24, 25]. There are no previous reports on the percentage of activated CD69 expressing NK cells with gestational age. 

Uterine NK cells play an important role in trophoblast invasion and spiral artery remodelling [4, 26]. Peripheral blood NK cells on the other hand are cytotoxic and an increase in number and activity has been associated with miscarriages and implantation failure [18, 27]. In our study, the pNK cells of pregnant women in the first trimester are reduced compared to nonpregnant women [28]. This was also observed by Kühnert et al. [23] and may be viewed as a logical adaption of the immune system to a healthy pregnancy. The precise role of uNK and pNK cells and their interrelationship in the later stages of pregnancy is unclear. As the pNK cells are regulated in the same way as the uNK cells, the increase in activated pNK cells though slight in terms of actual NK cell numbers is likely still significant and may reflect slightly raised antifetal maternal immune activity possibly in preparation for parturition. Although the CD56^bright^ pNK cells contribute less than 10% to the total pNK count, our study has demonstrated that it is mainly this type of pNK cells that become activated during pregnancy. However, while uNK cells show phenotypic and functional differences from pNK cells, changes in the numbers of the two subsets have been shown to be correlated by Park et al. [29] in women with recurrent miscarriage. Interestingly, CD56+ NK cells, bearing the natural cytotoxicity NKp46 receptor in the uterus and in the peripheral blood, have a similar cytokine profile [30]. The production of interferon-*γ* by uterine and peripheral blood NK cells after stimulation with soluble HLA G has also been shown to be similar [31]. Moreover, an NK1 type shift, the NK counterpart to Th1 cells, has been observed in the peripheral blood and uterine NKp46 cells in women with recurrent miscarriage [32]. Thus peripheral blood and uterine NK cells share certain important characteristics. As such an assessment of the state of activity of the peripheral blood NK cells provides a snap shot of uterine NK cell activity. 

Pregnancy is associated with a systemic inflammatory response [33–35] and with a significant involvement of interleukin 6 (IL-6) [36] and IL-33 [37]. The latter is gaining increasing importance in pregnancy and infertility as its receptor (ST2) is expressed by Th2 and NK cells [38]. Pregnancy is accompanied by an increase in the total number of leukocytes, mostly related to an increased neutrophil count [21, 22]. Additionally, there is a rise in the erythrocyte sedimentation rate and C-reactive protein [39, 40]. Such changes are observed in inflammation and sepsis and therefore, it is thought that the increase in the number of activated NK cells is in part related to the systemic inflammatory response that is evident in pregnancy. The growing mass of HLA mismatched tissue and circulating trophoblast tissue/debris are the additional components that contribute to the immunological response in pregnancy. Superimposed on the aforementioned processes are the decreasing circulating levels of tolerance promoting factors such as human chorionic gonadotropin that has very significant immunomodulating properties [41–44]. It is possible that the combined effect of these various factors may explain the increase in the activation of NK cells in pregnancy and possible maternal immune preparation for parturition. 

## 4. Conclusion

This study demonstrates the changes of peripheral blood NK cells and activated CD69 expressing NK cells in normal pregnancy, which may be used in the investigation of pathological singleton pregnancies in nulliparous women. The underlying cause of the changes in activated NK cells levels in the different stages of pregnancy remains unclear. 

## Figures and Tables

**Table 1:** Characteristics of the study population (median and range).

	First trimester (*n* = 45)	Second trimester (*n* = 46)	Third trimester (*n* = 42)
Gestational age in wks, median (range)	12^+6^ (11^+6^–13^+6^)	22^+0^ (20^+0^–22^+4^)	32^+1^ (31^+6^–33^+5^)
Maternal age in yrs, median (range)	29 (17–36)	28 (16–38)	29 (17–39)
Maternal weight in Kg on date of sampling, median (range)	68.4 (54.0–89.0)	69.0 (42.9–104.0)	76.0 (46.9–112.0)
BMI in Kg/m^2^ on date of sampling, median (range)	24.2 (18.0–32.7)	25.3 (17.1–36)	29.0 (17.4–42.3)
BMI in Kg/m^2^ at booking, median (range)	24.2 (18.0–32.7)	24.1 (17–34.9)	24.8 (13.5–40)
Racial origin			
Caucasian, *n* (%)	25 (58.1)	25 (54.3)	25 (56.8)
Afro-Caribbean, *n* (%)	18 (41.9)	21 (45.7)	19 (43.2)

**Table 2:** Mean (SD) total lymphocyte, natural killer cell, and CD69 counts in the study population.

	*N*	First trimester	*N*	Second trimester	*N*	Third trimester
Total lymphocyte count (×10^9^/L)						
Total, mean (SD)	45	1.93 (0.57)	46	1.68 (0.41)	42	1.79 (0.43)
Caucasian, mean (SD)	25	1.87 (0.44)	25	1.74 (0.44)	25	1.82 (0.47)
Afro-Caribbean, mean (SD)	20	1.99 (0.71)	21	1.60 (0.45)	17	1.74 (0.37)
NK cell count (×10^6^/L)						
Total, mean (SD)	45	232.6 (149.5)	46	183.4 (108.1)	42	166.9 (77.7)
Caucasian, mean (SD)	25	230.0 (130.1)	25	198.7 (126.1)	25	171.3 (85.3)
Afro-Caribbean, mean (SD)	20	236.0 (174.3)	21	165.1 (81.0)	17	160.4 (67.0)
NK cell/lymphocyte (%)						
Total, mean (SD)	45	11.6 (5.8)	46	10.8 (4.6)	42	9.3 (3.3)
Caucasian, mean (SD)	25	12.0 (5.3)	25	11.0 (4.8)	25	9.3 (3.1)
Afro-Caribbean, mean (SD)	20	11.3 (6.4)	21	10.5 (4.6)	17	9.2 (3.7)
CD69 count (×10^6^/L)						
Total, mean (SD)	30	1.88 (1.08)	30	2.14 (1.90)	30	2.56 (1.42)
Caucasian, mean (SD)	15	2.02 (1.25)	15	2.69 (2.48)	15	3.07 (1.50)
Afro-Caribbean, mean (SD)	15	1.75 (0.89)	15	1.59 (0.81)	15	2.05 (1.16)
CD69/NK cell (%)						
Total, mean (SD)	30	1.14 (0.76)	30	1.24 (0.72)	30	1.82 (1.29)
Caucasian, mean (SD)	15	1.03 (0.65)	15	1.45 (0.91)	15	1.98 (0.94)
Afro-Caribbean, mean (SD)	15	1.25 (0.86)	15	1.04 (0.39)	15	1.67 (1.59)

NK: natural killer.
